# Validation of a Measure of Subjective Well-Being: An Abbreviated Version of the Day Reconstruction Method

**DOI:** 10.1371/journal.pone.0043887

**Published:** 2012-08-27

**Authors:** Marta Miret, Francisco Félix Caballero, Arvind Mathur, Nirmala Naidoo, Paul Kowal, José Luis Ayuso-Mateos, Somnath Chatterji

**Affiliations:** 1 Instituto de Salud Carlos III, Centro de Investigación Biomédica en Red de Salud Mental, CIBERSAM, Madrid, Spain; 2 Department of Psychiatry, Hospital Universitario de La Princesa, Instituto de Investigación Sanitaria Princesa, Madrid, Spain; 3 Department of Psychiatry, Universidad Autónoma de Madrid, Madrid, Spain; 4 Dr. Sampurnanand Medical College, Jodhpur, Rajasthan, India; 5 Department of Health Statistics and Information Systems, World Health Organization, Geneva, Switzerland; 6 Research Centre for Gender Health and Ageing, Faculty of Health, University of Newcastle, Newcastle, New South Wales, Australia; Federal University of Rio de Janeiro, Brazil

## Abstract

**Background:**

The study of well-being is becoming a priority in social sciences. The Day Reconstruction Method (DRM) was developed to assess affective states. The aim of the present study was to validate an abbreviated version of the DRM designed for administration in population studies, and to assess its test-retest properties.

**Principal Findings:**

1560 adults from Jodhpur (India) were interviewed using an abbreviated version of the DRM, and a week later they were re-interviewed using the original long version of the DRM, after which the abbreviated version of the DRM was compared with the original version. A regression model considering interaction terms was employed to analyse the impact of sociodemographic characteristics on net affect. Test-retest reliability was assessed, and found to be moderate. Positive affect showed more test-retest reliability than negative affect, while net affect had more temporal stability than U-index. The affect of sets A, B, and C, taken together, had a moderate predictive ability compared with the affect obtained using the full version of the DRM: AUC = 0.67 for positive affect; 0.66 for net affect; 0.61 for negative affect; and 0.60 for the U-index. Household income, gender, and setting all had a significant impact on net affect.

**Conclusions:**

Net affect and positive affect showed moderate temporal stability, whereas negative affect and the U-index showed fair temporal stability. Evaluating the affective state using the abbreviated version of the DRM provides a profile of the population similar to that of the full version. The results provide considerable support for using the short version of the DRM as an instrument to measure subjective well-being in large population surveys.

## Introduction

Well-being is an emergent social and political priority. The Commission on the Measurement of Economic Performance and Social Progress recommended that economic measurement systems should shift emphasis from measuring economic production to measuring people’s well-being, and that information on the well-being of the population should be uniformly collected by every government [1]. However, this will only become possible if more population studies routinely include measurement of individual well-being as a prime objective [2].

Subjective well-being includes a person’s satisfaction with various domains of life, their overall judgement of life satisfaction, and their current affective state measured as a time-weighted metric of the amount of negative or positive emotions [3].

Overall judgement of life satisfaction is commonly measured by asking people a single question, such as, “Taking all things together, how would you say that you are these days: very happy, pretty happy, or not too happy?” Satisfaction with different domains of life can be assessed with instruments such as the World Health Organization Quality of Life assessment (WHOQOL) [4]. To assess the current affective state, Csikszentmihalyi and Larson [5] created the Experience Sampling Method, and later Kahneman, *et al*., [6] developed the Day Reconstruction Method (DRM) to assesses how people spend their time and how they experience the activities and settings of their lives. The DRM asks participants to systematically reconstruct their activities and experiences of the preceding day with procedures designed to reduce recall biases by inducing retrieval of the specifics of successive episodes.

The DRM has shown adequate psychometric properties. Krueger and Schkade [7] evaluated the test-retest reliability of the DRM by having the same respondents complete the questionnaire two weeks apart regarding the same day of the week. They found that both overall life satisfaction measures and affective experience measures derived from the DRM exhibited test-retest correlations ranging from 0.50 to 0.70. Dockray, *et al*., [8] evaluated the strength of associations between Ecological Momentary Assessment (EMA) (also known as the Experience Sampling Method) and DRM assessments of affect in the same individuals over the same time period, and found that the between-person correlations ranged from 0.58 to 0.90, concluding that the DRM provides reliable estimates of the intensity of affect and variations in affect over the day. The diurnal cycles of affect and tiredness produced by the Experience Sampling Method and the DRM are also remarkably similar [6], [9].

Bylsma, *et al*., [10] found high internal consistency, with multilevel reliability estimates higher than 0.90 for negative affect (NA) and positive affect (PA). They also computed pairwise Pearson’s correlations between average daily positive affect and negative affect with the DRM data and the ESM data regarding the same day, and these ratings were compared with a state measure of positive affect and negative affect reported when participants completed the DRM. All correlations were significant and large in magnitude (r = 0.62–0.72 for PA and 0.78–0.84 for NA).

Most of the studies performed so far to evaluate experienced well-being have been carried out with small, convenience samples due to the fact that the measures used– Experience Sampling Method and the original version of the Day Reconstruction Method –are time-consuming and costly. A study that has evaluated well-being in population samples is the Gallup World Poll [11]. In this survey, the well-being of representative samples of the adult population from 132 countries was assessed through telephone and door-to-door interviews. The affective state was assessed with questions about whether respondents experienced certain positive and negative feelings a lot during the previous day [11]. Nevertheless, the use of these self-report retrospective measures of well-being does not avoid memory and judgmental biases.

More recently, Krueger and Stone [12] designed the Princeton Affect and Time Survey (PATS), which is based on the DRM. In a telephone interview, respondents are first asked to describe each episode of the preceding day. Then, three episodes are randomly selected; for these episodes, respondents are asked a 5-minute module of questions, covering the extent to which they experienced six different emotions. Information on whether the individual was interacting with someone during the episodes is also collected. While more time-efficient, the limitations of this version are that it is administered by telephone, and that it only covers three episodes of the previous day.

An abbreviated version of the DRM that can be administered in population studies using face-to-face interviews was developed. The World Health Organization’s Study on global AGEing and adult health (SAGE) developed and tested such an abbreviated version. The aim of the present study was to validate this short version of the DRM by comparing the results with the original long version of the DRM and to evaluate its temporal stability.

## Materials and Methods

### Sample and Procedure

Probability sampling was employed to generate a random selection of older urban and rural respondents from Jodhpur (India) and the neighbouring area. Numbers of men and women were roughly equivalent, and an equal number of residents from urban and rural areas were interviewed.

A sample comprising 1560 adults (aged 18 years or older) was interviewed using the abbreviated version of the DRM. A week later (the same day of the week) they were re-interviewed with the original long version of the DRM. A mixture of weekday and weekend days (or work and non-work days) was obtained.

### Measures

The Day Reconstruction Method [6] was used to obtain information about participants’ daily activities and their subjective well-being. Through an interview, participants reconstructed their previous day’s activities, reported the positive and negative emotions associated with each activity and whom they were with, if anyone. The data provided a picture of the participants’ daily lives, including what they did and for how long, as well as a way of calculating how much of their time was spent feeling unpleasant emotions.

The abbreviated version of the DRM (available at http://www.who.int/healthinfo/systems/sage/en/index.html) was designed to last a maximum of 15 minutes in order to be used in general population surveys. Instead of reconstructing the full day, each participant reported only a portion of the previous day. This shortened version is composed of four different sets (A, B, C, and D), to which participants were randomly assigned. In sets A, B, and C, participants reconstructed only a portion of their previous day’s activities (starting with morning, afternoon, or evening, respectively) and responded to questions about each episode, including the nature of the activity (for example, working, shopping), people involved (alone, with spouse), and the extent to which they experienced various feelings–worried, rushed, irritated or angry, depressed, tense or stressed, calm or relaxed–and their level of enjoyment, on a scale ranging from 1 (not at all) to 3 (very much). In set D, participants reported the activities, people involved, and feelings for each part of the day (morning, afternoon, and evening) taken altogether, instead of activity by activity. In sets A, B, and C, the day was recorded in an event-by-event manner, whereas in set D it was recorded broadly according to what was done in the morning, afternoon, and evening.

A week later all the participants were interviewed with the original long version of the DRM, where participants reported the activities performed the previous day during the entire day in an event-by-event manner; that is, with the same detail as in sets A, B, and C, except starting from awakening in the morning and continuing through the full day.

At the end of the questionnaire in both interviews (baseline (short) and a week later (full)), there was a set of supplementary questions about whether respondents experienced certain positive and negative feelings a lot in the previous day (for example, “Did you feel …worried/sleepiness/bored… for much of the day yesterday? Yes or no”). Furthermore, interviewees were asked to compare themselves to other people of their age living in the same area and to say whether they are usually in a better, same or worse mood than most others, and whether they are more, same or less anxious than most others (for example, “Are you usually in a better mood or a worse mood than most others? Or are you about the same?”). In the baseline interview, participants were also asked to provide demographic information (age, gender, education level, marital status, household income). The questions were translated from English into Hindi, using a WHO translation and back-translation protocol. Ethical approvals from the Ethics Review Committee, World Health Organization and Ethics Review Committee, Dr SN Medical College were obtained as well as written informed consent from each participant.

### Statistical Methods

First, descriptive analyses of the sample were performed, and χ^2^ tests (for categorical variables) and ANOVA tests (for quantitative variables) were used to test differences between the sets.

The test-retest reliability of two types of measures of subjective well-being derived from the Day Reconstruction Method [6] was analysed: net affect and U-index. Net affect was defined as the average of the two positive emotions (calm/relaxed and enjoyment), minus the average of the five negative ones (worried, rushed, irritated/angry, depressed, and tense/stressed), resulting in positive affect minus negative affect. For sets A, B, and C, scores were weighed by activity duration. In set D, a raw score was calculated, because the affect items were not associated with single activities. Net affect scores ranged from −2 to 2. The U-index was defined as the proportion of time, aggregated over respondents, in which the highest rated feeling was a negative one. In set D, the U-index was not calculated because the duration of each activity was not collected.

The test-retest reliability was assessed using the Intraclass Correlation Coefficient (ICC). The 95% confidence interval for the ICC was calculated using the procedure based on Rosner’s approach using the *F*-test [13]. In order to quantify the reliability of the measures associated with continuous variables, it is more advisable to use the ICC [14], [15] than the Pearson product-moment correlation coefficient, since the test and retest scores could be highly correlated but show little agreement. ICC values were also reported for different groups based on education, household income, and setting.

The ICC represents the total variance in the measure (subject variability and measurement error) that was due to true differences between participants (subject variability). It accounts for the variability between, rather than within, the participants. Landis and Koch [16] suggested these standards for agreement levels: values in the interval (0, 0.2) were classified as poor, in the interval (0.2, 0.4) as fair, (0.4, 0.6) as moderate, (0.6, 0.8) as substantial, and (0.8, 1.0) as almost perfect. In the event of repeated measures, the ICC is large when there is little variation within the groups compared to variation among group means. A small ICC occurs when within-group variation is large compared with between-group variability, indicating that an unknown variable has introduced non-random effects in the different groups. The maximum value for the ICC is 1, and the minimum value is theoretically 0.

In order to assess test-retest reliability, in the full version of the DRM, the same time interval (for instance, the morning hours) was considered as for the activities reported in the corresponding short version DRM sets. Therefore, each set was compared with the analogous part in the full version of the DRM (that is, morning compared to morning, afternoon to afternoon, evening to evening). The reliability of positive and negative affect was also evaluated separately. Moreover, several paired *t*-tests were performed with the aim of comparing the mean scores in both administrations for the participants in each set, as well as to quantify the magnitude of the general bias produced between both evaluations. A measure of effect size, Cohen’s *d* corrected for paired *t*-tests [17], was reported in order to control the effects of the large sample size.

With the aim of testing whether affect at the population level was the same for sets A, B, and C as for the full day version, only participants who completed sets A, B or C at baseline were considered. Paired *t*-tests, evaluated whether mean scores in affect and U-index were different for the short and the long versions of the DRM. The affect at baseline was averaged over the population adding up the affect reported in sets A, B, and C, and then compared with the affect registered with the full version of the DRM at time 2. Several ROC-type analyses were carried out using the *nonbinROC* package [18] in *R* program [19], which implements nonparametric estimators proposed by Obuchowski [20] when the gold standard is measured on a continuous scale. Negative affect, positive affect, net affect, and U-index corresponding to the full version of the DRM were considered as gold standards. The area under the ROC curve (AUC) can be seen as a measure of similarity in measures obtained from the short versions and the full day version. Interpretation of the AUC is similar for binary and non-binary gold standards. The problem with these analyses carried out to assess the representativeness of sets A, B, and C together compared with the full version of the DRM is the day-to-day variation in affect, since both measures were taken one week apart. For this reason, similar analyses were carried out comparing the full version with the part of the second evaluation that corresponded to the same time period in sets A, B, and C, respectively. Analyses were carried out separately for each time period (morning, afternoon and evening), and also by summing the three time intervals.

Estimation of mean net affect associated with each activity was calculated, weighting the sample by the amount of time each participant spent in the corresponding activity. The percentage of respondents reporting each activity in each evaluation was also calculated. In order to assess the temporal stability of the affect associated with each activity, the Pearson product-moment correlation coefficient between the net affect associated with each activity in the test and in the retest was employed. Confidence intervals were calculated using Fisher’s transformations for correlation coefficients. In this correlation analysis, only activities reported at baseline by at least 5% of the sample were considered. These activities were ranked from the highest to the lowest net associated affect, and the correlation between the activities’ rankings on both evaluations was reported by means of Spearman’s correlation coefficient.

Considering the time of the first evaluation, systematic effects of three qualitative predictors on net affect were tested: education (coded as 0 = less than primary school, 1 = primary school completed or more), setting (0 = rural, 1 = urban) and household income (0 = first or second quintile of income (less wealthy), 1 = third, fourth or fifth quintile of income (more wealthy)). Age and sex (0 = female, 1 = male) were considered as covariates, and interaction terms among categorical variables were included in a first model. By means of an ANOVA test, an analysis was carried out to determine whether the model containing interaction terms explained an amount of variance significantly higher than the simplest model (without interaction terms).

The test-retest reliability for the dichotomous questions about how the respondent felt overall the day before the interview was estimated using the Delta and Kappa coefficients. Kappa is the most common measure of agreement and test-retest reliability for categorical data. Nevertheless, Kappa performs poorly when the marginal distributions are markedly asymmetrical: a high proportion of agreement can be drastically lowered by a substantial imbalance in the marginal total of the table, either vertically or horizontally [21]. Delta coefficient is not affected by this problem and refers to the total proportion of answers that are concordant (not by chance) [22], [23]. Kappa and Delta generally have very similar values, except when the marginal distributions are strongly unbalanced. Accuracy of the Delta model can be assessed by means of a Chi-square test for goodness of fit. Additionally, the test-retest reliability of the two questions was examined to determine how respondents compare themselves to other people regarding their mood and anxiety. Since the questions had a 3-point response option, the weighted Kappa coefficient was employed.

Confidence levels of 95% were considered in hypothesis tests. When significant differences appeared, effect sizes (Cramer’s *V* for χ^2^ tests, Cohen’s *f* for ANOVA tests, and Hedge’s *g* for unpaired *t*-tests) were reported. Statistical analyses were carried out using Stata version 11 [24] and R version 2.10.1 [19].

## Results

A total of 1560 people from Jodhpur (India) were interviewed. Table 1 presents the main characteristics of the sample. The percentage of participants who completed each of the four sets was 25%. The predominant religion was Hinduism (90.7% of the participants), with 99.7% of the sample belonging to a religious denomination. Significant differences among sets were found in terms of sex, age, and income quintile, although the small effect size (Cramer’s *V* = 0.21 for gender and 0.11 for income quintile; Cohen’s *f = *0.09 for age) indicates that they are probably due to the large sample size. Out of the initial sample, 22 participants (4 in set A, 9 in set B, 3 in set C, and 6 in set D) did not complete the second evaluation (full DRM). The main characteristics of these 22 participants (36.4% female, mean age = 56.2±17.6, 63.6% living in rural settings) were not significantly different from the sample as a whole.

**Table 1:** Demographic characteristics of participants who completed sets A, B, C, or D in baseline.

	Total	Set A	Set B	Set C	Set D	
	*n* = 1560	*n* = 390	*n* = 390	*n* = 390	*n* = 390	*p* *
**Female: n (%)**	829 (53.1)	166 (42.6)	169 (43.3)	234 (60.0)	260 (66.7)	<0.001
**Age, years: mean (SD)**	57.1 (17.6)	57.8 (17.6)	59.1 (16.7)	55.2 (18.1)	56.3 (17.6)	<0.01
**Highest education level completed: n (%)**						0.41
No formal education	791 (50.9)	188 (48.5)	193 (49.5)	197 (51.0)	213 (54.6)	
Less than primary school	106 (6.8)	26 (6.7)	32 (8.2)	22 (5.7)	26 (6.7)	
Primary school	232 (14.9)	62 (16.0)	55 (14.1)	50 (13.0)	65 (16.7)	
Secondary school	149 (9.6)	34 (8.8)	34 (8.7)	45 (11.7)	36 (9.2)	
High school	78 (5.0)	25 (6.4)	22 (5.6)	18 (4.7)	13 (3.3)	
College/university	120 (7.7)	28 (7.2)	33 (8.5)	34 (8.8)	25 (6.4)	
Post-graduate degree	78 (5.0)	25 (6.4)	21 (5.4)	20 (5.2)	12 (3.1)	
**Married or in partnership: n (%)**	1092 (73.1)	280 (74.6)	285 (75.6)	274 (73.3)	253 (68.7)	0.07
**Rural setting: n (%)**	692 (44.4)	164 (42.1)	176 (45.1)	191 (49.0)	161 (41.3)	0.12
**Income quintile: n (%)**						<0.001
1 (Lowest)		55 (14.1)	85 (21.8)	102 (26.2)	70 (18.0)	
2		84 (21.5)	79 (20.3)	69 (17.7)	80 (20.6)	
3		85 (21.8)	70 (18.0)	67 (17.2)	89 (22.9)	
4		84 (21.5)	63 (16.2)	62 (15.9)	103 (26.5)	
5 (Highest)		82 (21.0)	93 (23.9)	89 (22.9)	47 (12.1)	

* *p*-value associated to differences among sets using χ^2^ test (categorical variables) or ANOVA test (quantitative variables).

Mean time of administration (considering the DRM and the supplementary questions) was lower in the short version than in the long version (16.4±8.5 vs. 29.6±18.1 minutes). Considering only the short version, the time of administration of set D (14.9±6.0 minutes) was slightly lower than that of the other sets (17.8±5.9 minutes in set A, 16.8±10.3 in set B, and 17.2±10.1 in set C).

In general, the test-retest reliability of the measures obtained from the DRM was moderate. As can be seen in Table 2, the ICC comparing the same time period on the test and the retest showed values slightly lower for set A on all the measures. Positive affect was clearly more reliable than negative affect and U-index. The highest ICC value was found for positive affect in set D. However, ICC values corresponding to test-retest reliability of net affect were similar in sets B, C, and D. When pooling the four sets across the entire sample, the results showed better test-retest reliability for positive affect, followed by net affect. Negative emotion measures, negative affect and U-index, presented fair test-retest reliability.

The ICC based on education, household income, and setting can be seen in Table S1. The reliability in the affect measures was slightly higher for people with less than primary education, those living in rural areas, and those with low income.

**Table 2:** Mean±SD of affect measures for each set and overall in the test and the retest. *p*-values associated to paired *t*-tests and ICC values.

	Test	Retest				
	Mean±SD	Mean±SD	*t*	*p*	*d*	ICC (95% CI)
**Set A (n = 386)**						
Net affect	1.10±0.61	1.08±0.50	0.59	0.56		0.34 (0.25,0.43)
PA	2.26±0.43	2.21±0.39	2.47	0.01	0.14	0.41 (0.33,0.49)
NA	1.17±0.31	1.13±0.23	2.19	0.03	0.13	0.34 (0.25,0.42)
U-index	0.22±0.35	0.20±0.30	1.54	0.12		0.33 (0.24,0.41)
**Set B (n = 381)**						
Net affect	1.08±0.63	1.07±0.52	0.25	0.81		0.43 (0.34,0.51)
PA	2.22±0.46	2.18±0.41	2.11	0.04	0.11	0.49 (0.41,0.57)
NA	1.14±0.29	1.11±0.21	2.50	0.01	0.15	0.26 (0.17,0.35)
U-index	0.23±0.36	0.14±0.26	4.55	<0.001	0.28	0.26 (0.17,0.35)
**Set C (n = 387)**						
Net affect	0.96±0.65	0.97±0.53	−0.25	0.8		0.47 (0.39,0.56)
PA	2.13±0.47	2.08±0.41	2.16	0.03	0.11	0.49 (0.41,0.56)
NA	1.17±0.27	1.12±0.21	3.67	<0.001	0.22	0.28 (0.18,0.37)
U-index	0.29±0.37	0.21±0.31	4.07	<0.001	0.24	0.33 (0.23,0.42)
**Set D (n = 384)**						
Net affect	1.02±0.68	1.09±0.48	−2.25	0.03	0.11	0.43 (0.34,0.52)
PA	2.19±0.49	2.17±0.39	0.85	0.39		0.53 (0.46,0.60)
NA	1.18±0.31	1.09±0.17	5.86	<0.001	0.35	0.27 (0.18,0.36)
**4 sets pooled (n = 1538)**						
Net affect	1.04±0.64	1.05±0.51	−0.83	0.41		0.42 (0.38,0.46)
PA	2.20±0.46	2.16±0.40	3.82	<0.001	0.10	0.49 (0.46,0.52)
NA	1.16±0.30	1.11±0.20	7.07	<0.001	0.21	0.28 (0.24,0.33)
U-index*	0.25±0.36	0.18±0.29	5.89	<0.001	0.20	0.31 (0.26,0.36)

Note: PA = Positive affect;

NA = Negative affect.

* Considering only sets A, B, and C.

The paired *t*-tests showed significant differences in positive and negative affect between the first and second evaluation in sets A, B, and C (Table 2). However, the effect sizes associated were in general lower than 0.20 and did not meet the standard of a small effect size, suggesting that the statistical significance was due to the large sample size more than to evidence of change in scores over time. Similar results were found in set D based on the net affect, but the low effect size (*d* = 0.11) shows the invariance of scores on net affect. On the other hand, differences found in set D for negative affect were significant, with moderate effect size (*d* = 0.35). In terms of the U-index, significantly lower scores were found on the retest for sets B and C, with a small associated effect size.

Comparing mean scores of sets A, B, and C pooled together, and the long version, lower mean scores were observed on the long version for positive affect (2.21±0.46 vs. 2.14±0.37; *t* (1153) = 5.45; *p*<0.001; *d* = 0.16) and negative affect (1.16±0.29 vs. 1.12±0.21; *t* (1154) = 5.27; *p*<0.001; *d* = 0.18), although the effect sizes were very low. On the other hand, significant differences were not found in terms of net affect (1.04±0.63 vs. 1.02±0.48; *t* (1153) = 1.28; *p* = 0.20) and U-index (0.25±0.36 vs. 0.23±0.32; *t* (1152) = 1.75; *p* = 0.08).

The affect results of sets A, B, and C taken together and aggregated over respondents at baseline, had a moderate predictive ability for scores on the same measures obtained in the full version of the DRM a week later. Higher values were found for positive affect (AUC = 0.67, s.e. = 0.01) and net affect (AUC = 0.66, s.e. = 0.01), while negative affect measures had a lower associated AUC value: 0.61 (s.e. = 0.01) for negative affect, and 0.60 (s.e. = 0.01) for the U-index. Similar values were obtained in set D: AUC = 0.66 (s.e. = 0.02) for net affect, AUC = 0.64 (s.e. = 0.01) for positive affect, and AUC = 0.61 (s.e. = 0.01) for negative affect.

When comparing the full version of the DRM with the part of the second evaluation corresponding to the same time interval as sets A, B, and C, respectively, AUC values indicated that each portion of the day can be considered fairly representative of the full day in this population (Table 3). Considering participants independently from sets A, B or C, the highest AUC values were found for net affect and positive affect. Values higher than 0.90 indicate that of two randomly chosen scores, there is more than a 90% chance that the highest score on the short version of the DRM will have a higher score on the full version than the lowest one.

**Table 3:** AUC values (s.e.) corresponding to ROC analyses comparing the morning, afternoon, and evening portions of the second evaluation with the full day in the same evaluation.

	From wake-up onwards	From noon onwards	From 6pm onwards	Global
Net affect	0.87 (0.01)	0.94 (0.01)	0.94 (0.01)	0.92 (0.01)
Positive affect	0.86 (0.01)	0.93 (0.01)	0.94 (0.01)	0.91 (0.01)
Negative affect	0.82 (0.01)	0.85 (0.01)	0.87 (0.01)	0.85 (0.01)
U-index	0.79 (0.01)	0.78 (0.01)	0.80 (0.01)	0.79 (0.01)

Furthermore, high correlations were found between negative affect and U-index in participants from sets A, B, and C: *r* = 0.82; 95% CI = (0.80,0.84) in the first evaluation, and a similar value, *r* = 0.77; 95% CI = (0.75,0.80), on the second evaluation.

*Eating*, *resting,* and *chatting with someone* were the most reported activities, with more than half of the sample reporting them on the test and on the retest. On both evaluations, *religious activity*, *reading,* and *exercising or leisurely walk* had the highest associated mean net affect, whereas the lowest net affect was associated with *doing housework*, *preparing food,* and *watching children*. The correlation coefficient values between the net affect on the test and retest were higher for activities like *religious activity*, *eating*, *resting*, *preparing food,* and *working*. On the other hand, according to the results shown in Table 4, no significant correlation was found between the net affect scores obtained on both evaluations for *walking somewhere*, *exercising or leisurely walk,* and *watching TV*. Spearman’s correlation coefficient between the rankings of activities on both evaluations was 0.90, 95% CI = (0.70, 0.97), while Pearson’s correlation coefficient of mean net affect across activities was 0.96, 95% CI = (0.86, 0.99).

**Table 4:** Activities coded in the baseline DRM ranked from the highest to the lowest weighted-duration mean net affect_._

*Activities reported by at least 5% of* *the sample in baseline*	Test		Retest		*n_test-retest_ (%)*	*r* (95% CI)	
	%	Mean net affect (s.e.)	%	Mean net affect (s.e.)			
Religious activity	30.6	1.43 (0.04)	51.7	1.36 (0.04)	244 (21.1)	0.41 (0.30, 0.51)	
Reading	10.0	1.40 (0.11)	19.9	1.46 (0.07)	61 (5.3)	0.25 (0.01, 0.48)	
Exercising or leisurely walk	8.7	1.36 (0.08)	19.6	1.39 (0.06)	57 (4.9)	0.08 (−0.18, 0.33)	
Grooming or bathing (self)	31.2	1.18 (0.04)	82.3	1.16 (0.04)	328 (28.4)	0.27 (0.17, 0.37)	
Watching TV	20.0	1.17 (0.04)	40.9	1.10 (0.03)	170 (14.7)	0.08 (−0.07, 0.23)	
Chatting with someone	58.0	1.11 (0.03)	81.5	1.05 (0.02)	578 (50.1)	0.31 (0.23, 0.38)	
Working	25.7	1.09 (0.05)	42.7	0.96 (0.03)	209 (18.1)	0.34 (0.22, 0.46)	
Eating	72.9	1.07 (0.02)	95.7	1.11 (0.02)	809 (70.1)	0.40 (0.34, 0.46)	
Walking somewhere	15.3	1.02 (0.07)	24.4	1.06 (0.06)	96 (8.3)	0.01 (−0.19, 0.21)	
Rest	65.0	1.00 (0.04)	95.5	0.99 (0.02)	726 (62.9)	0.39 (0.33, 0.45)	
Doing housework	32.9	0.96 (0.05)	53.4	0.92 (0.03)	271 (23.5)	0.23 (0.12, 0.34)	
Preparing food	16.0	0.91 (0.06)	23.7	0.93 (0.04)	138 (12.0)	0.38 (0.22, 0.51)	
Watching children	12.2	0.84 (0.17)	24.3	0.86 (0.07)	69 (6.0)	0.31 (0.08, 0.51)	
***Rest of activities***	**%**	**Mean net affect (s.e.)**	**%**	**Mean net affect (s.e.)**	***n_test-retest_*** **(%)**		
Intimate relations/sex	0.1	1.50 (−)	0.1	1.00 (−)	0		
Listening to the radio	2.0	1.05 (0.19)	5.5	1.06 (0.10)	8 (0.7)		
Other leisurely activity	3.4	1.00 (0.21)	10.6	1.25 (0.06)	6 (0.5)		
Playing	0.6	1.44 (0.22)	1.1	1.13 (0.17)	0		
Providing care to someone	2.1	0.65 (0.36)	5.1	0.68 (0.17)	2 (0.2)		
Shopping	1.7	1.31 (0.18)	5.3	1.04 (0.08)	4 (0.4)		
Subsistence farming	1.8	0.41 (0.38)	6.2	0.66 (0.08)	8 (0.7)		
Travelling by bicycle	0.1	−0.30 (−)	0.3	1.22 (0.68)	0		
Travelling by car/bus/train	0.2	0.68 (0.14)	0.7	1.16 (0.16)	0		

Descriptive statistics correspond to activities reported in the test and the retest by 1154 participants who completed sets A, B, and C in baseline as well as the long version a week later.

Note: % = Percentage of the sample reporting the activity.

n_test-retest_ = Number of participants (%) who mentioned the activity in the test and in the retest_._

*r* = Pearson correlation coefficient between net affect values in the test and in the retest.

Confidence intervals for the correlations are not symmetric because they are based on the non-linear Fisher’s *z* transformation (*z* = 0.5[ln(1+*r*)−ln(1−*r*)]), which is normally distributed and used for significance testing.

The ranking was carried out for activities reported by at least 5% of the sample in baseline. The rest of activities were sorted alphabetically.

Few people mentioned the other activities coded on the DRM, and this fact makes it difficult to draw conclusions. Taking into account the lower levels of endorsement of these other activities, it could be observed that activities such as *subsistence farming* or *providing care to someone* have a lower associated net affect than, for example, *shopping*, *listening to the radio,* and *other leisurely activity* (Table 4).

Income level, setting and sex were found to be significant predictors of net affect. Being male, with a high income, and living in an urban area were associated with a higher net affect. Interactions between education and sex, and between income and sex, were both significant. The significant interaction terms showed that the effect of education and household income on net affect was different for men and women, being stronger for women. The variance explained by this initial model was significantly higher than the variance explained by the model without the interaction terms (*F* (6, 1535) = 3.50; *p*<0.01). The final regression model, considering only the significant interaction terms in the initial model, was significant, although it did not explain a large amount of variance (*F* (7, 1539) = 27.66, *p*<0.001; adjusted *R*-squared = 0.11). The results of this regression model are shown on Table S2.

The Delta coefficient value was employed to assess the test-retest reliability for the 14 additional dichotomous questions corresponding to the day before the interview. In each case, the goodness of fit of the Delta model was adequate, since the Chi-square test was not significant. The overall agreement given by the Delta coefficient was substantial (and larger than what Kappa would indicate) in most cases (see Table S3). The Delta coefficient value was moderately low only for *worried*, *physical pain*, *headache,* and *smile*. For *physical pain* and *smile*, the value was similar to the one obtained with Kappa, since in both cases the marginal distributions are not very unbalanced, the proportion of “yes” is not very different from the proportion of “no”. In general, the test-retest reliability was more adequate for the two questions about anxiety and mood than for the general questions about how the person felt the previous day.

## Discussion

One of the strengths of the present study is that its design makes it possible to compare abbreviated versions of the DRM with the original, longer one, and to test its temporal stability. Compared with other instruments for evaluating well-being, the DRM has the advantage of reducing memory and judgmental biases. Furthermore, recording the day in an event-by-event manner allows the evaluation of time use and the emotional state associated with each activity. Regarding the feasibility of use, the advantage of this shorter version is that it is possible to evaluate experienced well-being in large population surveys that use a face-to-face administration mode and does not require the use of sophisticated devices as the ESM does [5]. Furthermore, it does not pose a big respondent burden in terms of interview time. The abbreviated version of the DRM was designed to last a maximum of 15 minutes, and together with the supplementary questions it lasted around 16 minutes, whereas completion times for the long version were approximately twice that. This long version is also shorter than the self-administered original DRM instrument [6], which ranged from 45 to 75 minutes [6], [7]. Additionally, this version of the questionnaire, rotated across respondents, has the advantage that it can be administered to people regardless of their education level. Despite more than half of the sample having received little or no formal education, the DRM was administered successfully.

The reliability coefficients calculated on the test-retest were statistically significant, although the test-retest reliability was modest for some measures. Combining the test-retest reliability and the invariance of mean scores in the test-retest, the net affect is the most reliable measure. On the other hand, positive affect appears to be more reliable than negative affect, obtaining results similar to those reported by Krueger and Schkade [7]. Even though day-to-day variations exist in the activities people perform and in the affect experienced, the measurement of affect is relatively stable at the population level. People living in rural areas, with a low household income, and with a low level of education had more stable affect over time. On the other hand, men, people from urban areas, and those with higher income had a higher net affect. Previous studies have also shown a relationship between household income and net affect, although the correlation is higher for life satisfaction [6], [7].

The affect scores of the pooled sets A, B, and C together, predicted the scores obtained in the full version of the DRM with a moderate degree of precision. The AUC values obtained from the ROC analyses provide evidence of excellent accuracy of the short versions being representative, in terms of emotions, of the full day. These results are indicative of criterion validity of the short versions of the DRM, using the measures associated with emotions reported in the full version as the gold standard.

While no variations were found in positive and negative affect by days of the week (detailed results not shown; available from the authors upon request), as reported in previous studies, this is due to the fact that in the study population a large proportions of respondents had been working on Saturdays and Sundays with no clear distinctions to define a ‘weekend’. However, comparing positive and negative affect in respondents who had worked the previous day with those who hadn’t, the latter group had significantly higher positive affect and lower negative affect scores mimicking the ‘weekend’ effect reported in other studies.

The high correlation between the net affect at baseline and in the retest and between the rankings of activities on both evaluations show that a given activity produces a similar average experience at different moments at the population level. This finding has also been previously reported in other studies [7]. Nevertheless, compared with the results in other studies, the affect associated with some activities was different, which can be explained by cultural and other differences found in the samples. *Religious activity*, *reading*, and *exercising or leisurely walk* showed the highest net affect in this study, whereas *doing housework*, *preparing food* and *watching children* showed the lowest. Religious activities appear to elicit different ranges of emotions in Western studies [6], [7], [25], sometimes ranking below other activities such as relaxing or doing exercise. This might be explained by the cultural differences in the samples; the fact that in this study 99.7% of the sample identified themselves with a religion is an indicator of its relevance in India. Furthermore, *working* appeared higher in the ranking than in other studies [6], [7], [25], while *cooking* had a net associated affect lower than in other studies [6], [7], [25].

Regarding the coding of the activities, there are some activities that were reported by only a few participants. *Travelling* (both by bicycle and by car/bus/train), and *intimate relations* were reported by less than 1% of the sample both at baseline and in the retest. Depending on the purpose of the study, in the future it might be useful to include *travelling* in a broader category. The activity *intimate relations* is usually one of the least reported [6], [7] and it might be considered whether to code it as an independent activity, especially if the DRM is interviewer administered, when it might be highly underreported.

Regarding the differences between the use of sets A, B, C (randomly assigning the participants to the morning, afternoon or evening sets), and set D, the results showed that all of the different versions had moderate predictive ability over the full version of the DRM. Although set D is significantly shorter, A, B, and C together provide relevant information about the feelings associated with each activity that is missing in set D.

As expected, the temporal stability of the questions about mood and anxiety in general was higher than the overall questions about how the respondent felt the day before the interview. Nevertheless, all of them showed a temporal stability between moderate and high.

### Conclusions

While net affect and positive affect showed moderate temporal stability, negative affect and the U-index showed slightly lower temporal stability. Positive affect is more stable over time than negative affect. It is unclear what factors may have contributed to this finding of differences in the replicability of positive vs. negative affect in our study. Further exploration will be required to determine the underlying reasons for these differences such as individual temperament, cultural acceptability of talking about negative emotions and the amount of time spent in these different emotive states over consecutive days in individuals. Nonetheless, evaluating affective states with the abbreviated version of the DRM aggregated over the population, combining the morning, afternoon, and evening sets, provides a similar profile of the population than administering the full day version to all the respondents. The results provide considerable support for the use of the short version of the DRM as an instrument to measure subjective well-being in large population surveys.

## Supporting Information

Table S1 Intraclass correlation coefficient (95% CI) between the test and retest evaluations in the affect measures by education, income, and setting (n = 1538).
(DOCX)

Table S2 Final equation for the linear regression analysis: impact of education, quintile of income, and setting over net affect, controlling for sex and age.
(DOCX)

Table S3 Test-retest reliability for the general questions about feelings on the day before the interview and for two questions about anxiety and mood.
(DOCX)
