# Supplementary material for: Validation of a Measure of Subjective Well-Being: An Abbreviated Version of the Day Reconstruction Method
Source: PLoS One. 2012 Aug 27;7(8):e43887. doi: 10.1371/journal.pone.0043887 (PMC3428291; doi:10.1371/journal.pone.0043887)
Supplement: Table S1 — Intraclass correlation coefficient (95% CI) between the test and retest evaluations in the affect measures by education, income, and setting (n = 1538). (DOCX) [file pone.0043887.s001.docx]

**Supporting Information**

**Table S1**. Intraclass correlation coefficient (95% CI) between the test and retest evaluations in the affect measures by education, income, and setting (n = 1538).

|  | **Net affect** | **Positive affect** | **Negative affect** | **U-index** |
| --- | --- | --- | --- | --- |
| **Education** |  |  |  |  |
| Less than primary school | 0.44 (0.39,0.49) | 0.51 (0.46,0.56) | 0.30 (0.24,0.36) | 0.35 (0.27,0.43) |
| Primary school completed or higher | 0.35 (0.28,0.41) | 0.43 (0.36,0.49) | 0.23 (0.16,0.30) | 0.21 (0.13,0.29) |
| **Setting** |  |  |  |  |
| Rural area | 0.39 (0.33,0.45) | 0.48 (0.42,0.53) | 0.26 (0.19,0.32) | 0.33 (0.25,0.40) |
| Urban area | 0.36 (0.30,0.41) | 0.43 (0.37,0.48) | 0.29 (0.23,0.35) | 0.22 (0.14,0.29) |
| **Household income** |  |  |  |  |
| Low (1^st^ or 2^nd^ quintile) | 0.44 (0.38,0.51) | 0.51 (0.45,0.57) | 0.27 (0.20,0.35) | 0.34 (0.26,0.42) |
| High (3^rd^-5^th^ quintile) | 0.34 (0.28,0.40) | 0.42 (0.37,0.48) | 0.27 (0.21,0.33) | 0.22 (0.15,0.29) |
