# Supplementary material for: Validation of a Measure of Subjective Well-Being: An Abbreviated Version of the Day Reconstruction Method
Source: PLoS One. 2012 Aug 27;7(8):e43887. doi: 10.1371/journal.pone.0043887 (PMC3428291; doi:10.1371/journal.pone.0043887)
Supplement: Table S2 — Final equation for the linear regression analysis: impact of education, quintile of income, and setting over net affect, controlling for sex and age. (DOCX) [file pone.0043887.s002.docx]

**Supporting Information**

Table S2: Final equation for the linear regression analysis: impact of education, quintile of income, and setting over net affect, controlling for sex and age.

| **Model** | **Estimate (s.e.)** | **t** | ***p*> \|t\|** |
| --- | --- | --- | --- |
| (Constant) | 0.70 (0.07) | 10.30 | <0.001 |
| Education (Ref. = Less than primary school) | 0.10 (0.06) | 1.73 | 0.08 |
| Household income (Ref. = 1^st^ or 2^nd^ quintile) | 0.19 (0.05) | 3.54 | <0.001 |
| Setting (Ref. = Rural area) | 0.29 (0.04) | 6.71 | <0.001 |
| Sex (Ref. = Female) | 0.33 (0.05) | 6.46 | <0.001 |
| Age | -0.00 (0.00) | -0.89 | 0.37 |
| Sex*Education | -0.13 (0.07) | -1.82 | 0.07 |
| Sex*Income | -0.16 (0.07) | -2.25 | 0.03 |

Ref. = Reference category
