# Supplementary material for: Validation of a Measure of Subjective Well-Being: An Abbreviated Version of the Day Reconstruction Method
Source: PLoS One. 2012 Aug 27;7(8):e43887. doi: 10.1371/journal.pone.0043887 (PMC3428291; doi:10.1371/journal.pone.0043887)
Supplement: Table S3 — Test-retest reliability for the general questions about feelings on the day before the interview and for two questions about anxiety and mood. (DOCX) [file pone.0043887.s003.docx]

**Supporting Information**

**Table S3**. Test-retest reliability for the general questions about feelings on the day before the interview and for two questions about anxiety and mood.

|  | **Agreement (%)** | **Expected agreement (%)** | ***k* (95% CI)** | **Δ (s.e.)** |
| --- | --- | --- | --- | --- |
| Worried | 74.8 | 61.1 | 0.35 (0.30,0.40) | 0.50 (0.02) |
| Rushed | 88.3 | 86.2 | 0.16 (0.08,0.23) | 0.77 (0.02) |
| Irritated/angry | 83.9 | 78.8 | 0.24 (0.17,0.31) | 0.68 (0.02) |
| Depressed | 83.8 | 74.0 | 0.38 (0.32,0.44) | 0.68 (0.02) |
| Tense/stressed | 83.1 | 72.7 | 0.38 (0.32,0.44) | 0.67 (0.02) |
| Relaxed | 82.4 | 73.0 | 0.35 (0.29,0.41) | 0.65 (0.02) |
| Enjoying | 81.1 | 69.3 | 0.39 (0.33,0.44) | 0.62 (0.02) |
| Lonely | 85.6 | 79.8 | 0.29 (0.22,0.36) | 0.71 (0.02) |
| Bored | 89.0 | 81.7 | 0.40 (0.33,0.47) | 0.78 (0.02) |
| Physical pain | 69.7 | 51.6 | 0.38 (0.33,0.42) | 0.40 (0.02) |
| Sleepiness | 87.5 | 82.4 | 0.29 (0.21,0.36) | 0.75 (0.01) |
| Stomach ache | 84.3 | 77.3 | 0.31 (0.24,0.37) | 0.70 (0.02) |
| Headache | 72.3 | 61.3 | 0.29 (0.23,0.34) | 0.45 (0.02) |
| Smile/laugh | 71.5 | 51.1 | 0.42 (0.37,0.46) | 0.43 (0.02) |
|  | **Agreement (%)** | **Expected agreement (%)** | ***k_w_* (95% CI)** | |
| Mood | 88.4 | 79.0 | 0.45 (0.40, 0.50) | |
| Anxiety | 86.3 | 76.6 | 0.42 (0.37, 0.47) | |

*k* = Kappa coefficient; Δ = Delta coefficient; *k_w_* = Weighted Kappa coefficient
